# Acupotomy therapy for lumbar disc herniation: Protocol for a systematic review and meta-analysis

**DOI:** 10.1097/MD.0000000000012624

**Published:** 2018-10-05

**Authors:** Cai Jiang, Yinyan Li, Miaomiao Guo, Xiaomei Li, Jinhua Guo, Shengxian Yu, Zhonghua Lin

**Affiliations:** a Fujian Provincial Hospital, Fuzhou; b Fujian University of Traditional Chinese Medicine, Fujian, China.

**Keywords:** acupotomy, lumbar disc herniation, protocol, systematic review

## Abstract

**Background:**

Lumbar disc herniation (LDH) is a clinically common degenerative spinal disease that adversely affects patients’ quality of life and work ability. Although several articles have reported that acupotomy can improve the clinical symptoms of LDH, the efficiency of acupotomy has not been scientifically or methodically evaluated. The primary objective of this study is to evaluate and synthesize evidence on the effectiveness and safety of acupotomy therapy in patients with LDH.

**Method:**

We plan to search eight English and Chinese electronic databases, including the Web of Science, Cochrane Library, PubMed, EMBASE, SinoMed, Wanfang, China Science and Technology Journal (VIP), and China National Knowledge Infrastructure (CNKI) databases, for potentially eligible studies. Randomized controlled trials (RCTs) on acupotomy treatment in LDH patients will be searched for independently by 2 reviewers in the databases from their inception to August 2018. We will combine data from clinically homogenous studies in a fixed effect meta-analysis using RevManV.5.3.5, and the evidence level will be assessed by using the method for Grading of Recommendations Assessment, Development, and Evaluation (GRADE).

**Results:**

This systematic review and meta-analysis will use high-quality evidence-based medicine to assess the efficacy and safety of acupotomy therapy in LDH.

**Conclusion:**

This study protocol will provide strong evidence to determine whether acupotomy therapy is an effective and safe intervention for patients with LDH.

## Abstract 2

Supplemental Digital Content is available in the text

## 1 Introduction

Lumbar disc herniation (LDH) is one of the most common degenerative spinal disorders in young and middle-aged patients.^[1]^ LDH is highly prevalent among patients aged 30 to 50 years, and approximately 95% of lumbar disc herniations occur at the L4/L5 and L5/S1 levels.^[2]^ The main symptoms of LDH include lower back pain, radiation pain on one side or in the lower extremities, weakening of muscle strength, and sensory disturbances.^[3,4]^ Sciatica is usually associated with lumbar disc herniation, with a lifetime prevalence of more than 30%.^[5]^ In addition, the symptoms of LDH can progress to a more serious stage, even leading to muscular atrophy, motor disturbances, and urinary and faecal incontinence, which adversely affect patients’ quality of life and ability to work.^[6,7]^

Many therapeutic interventions have been proposed for the treatment of LDH, including non-invasive treatments (conservative treatment), minimally invasive percutaneous injections (non-vascular intervention), and surgery.^[8,9]^ Non-operative treatments, including non-steroidal anti-inflammatory drugs, physical therapy, acupuncture, exercises, and epidural steroid injections, are feasible in clinical practice and are favoured by the majority of patients because of their effectiveness and low risk of complications.^[6,7,10,11]^ However, among the many non-operative treatments, there is no consensus on which is the optimal management strategy for LDH patients.^[12]^

Compared with conservative treatment, surgical intervention may have greater improvements or faster rates of pain relief.^[13,14]^ Despite significant improvements in surgical techniques, complications such as recurrent lumbar disc herniation do occur. The recurrence rate has been reported to vary between 5% and 18%,^[15]^ and reoperations lead to physical and psychological suffering for patients as well as substantial costs for society.^[16,17]^

Acupotomy was developed by Zhu Hanzhang in 1976, and the surgical tool consists of a handle, needle body and blade.^[18]^ Acupotomy therapy is considered a minimally invasive surgery that uses traditional Chinese medicine and combines Chinese acupuncture therapy and modern surgical principles.^[19]^ Clinicians of traditional Chinese medicine, orthopedics, and pain departments have widely used acupotomy in the treatment of skeletal muscle diseases in China with satisfactory efficacy.^[18,20,21]^ In addition, some clinical studies on lumbar disc herniation have reported pain relief and functional recovery after acupotomy.^[22,23]^ In the treatment of lumbar disc herniation, the role of acupotomy is to reduce nerve pressure, improve peripheral blood circulation, and restore the kinetic state of soft tissue.^[1,24]^

In this study, evidence-based medicine will be used to analyse and evaluate clinical randomized controlled trials (RCTs) on the use of acupotomy therapy for LDH. To the best of our knowledge, this systematic review and meta-analysis is the first to assess the effectiveness and safety of acupotomy therapy for LDH. We strongly believe that a well-designed systematic review and meta-analysis is quite important to provide convincing evidence about the effectiveness and safety of acupotomy therapy for further enhancing the clinical curative effect on patients with LDH.

## 2 Methods

### 2.1 Design and registration of the review

This systematic review and meta-analysis protocol has been registered in the international prospective register of systematic reviews (https://www.crd.york.ac.uk/PROSPERO/#myprospero). The registration number is CRD42018096447. This systematic review protocol is structured in accordance with the Preferred Reporting Items for Systematic Reviews and Meta-analyses Protocols (PRISMA-P) statement guidelines.^[25]^ Since this is a literature-based study, there is no requirement of ethical approval for this protocol.

### 2.2 Inclusion criteria for study selection

#### 2.2.1 Type of study

We will screen the research literature according to the criteria of the review objectives and participants, interventions, comparisons, outcomes (PICO). All RCTs on the use of acupotomy therapy in LDH will be included in this systematic review and meta-analysis. Review articles, animal studies, non-clinical studies, and case reports will be excluded. In addition, the language of the publications will be limited to Chinese and English.

#### 2.2.2 Types of participants

Studies will have recruited eligible subjects who have been diagnosed with lumbar herniated discs by CT or magnetic resonance imaging (MRI) within the past 5 years and who are older than 18 years old. Studies that include patients with specific or systematic diseases (such as blood disease, spinal tumours, cauda equina syndrome, and fractures/joint dislocations) or pregnancy will be excluded.

#### 2.2.3 Types of interventions

The review will comprise clinical trials that focus on acupotomy treatment. There will be no limitations in terms of the needle materials, frequency of treatment, treatment methods, and treatment courses. The control group will adopt internationally recognized therapies such as drug therapy, block therapy, surgery, or no treatment, and acupuncture will also be included.

#### 2.2.4 Types of outcome measures

The primary outcomes of interest will include improvement rates, functional tests, and pain relief. The secondary outcomes of interest will consist of recurrence rates, quality of life, muscle thickness, and adverse events, such as haemorrhage, serious discomfort, abscess, subcutaneous nodules, and infection.

### 2.3 Data sources

The main sources of information that will be obtained in this study include electronic resource databases, trial registries, retroactive references, and different types of grey literature.

We plan to search eight English and Chinese electronic databases, including the Web of Science, Cochrane Library, PubMed, EMBASE, SinoMed, Wanfang, China Science and Technology Journal (VIP), and China National Knowledge Infrastructure (CNKI) databases, for potentially eligible studies. Reference lists of the relevant literature and systematic reviews, as well as the tables of contents related to lumbar disc herniation and acupotomy will also be searched. RCTs on acupotomy treatment in LDH patients will be searched for independently by 2 reviewers in the databases from their inception to August 2018.

### 2.4 Search strategy

The strategy will be created according to the Cochrane handbook guidelines. The search keywords or combination subject terms will include lumbar disc herniation, herniated disk, disc prolapse, slipped disc needle knife, small needle knife, acupotomy, and randomized controlled trials. Equivalent search words will be used in the Chinese databases. The detailed strategies for searching the PubMed database will be presented in Appendix 1. For Chinese databases, these searching terms will be accurately translated.

### 2.5 Data collection and analysis

#### 2.5.1 Selection of studies

**Figure 1:**
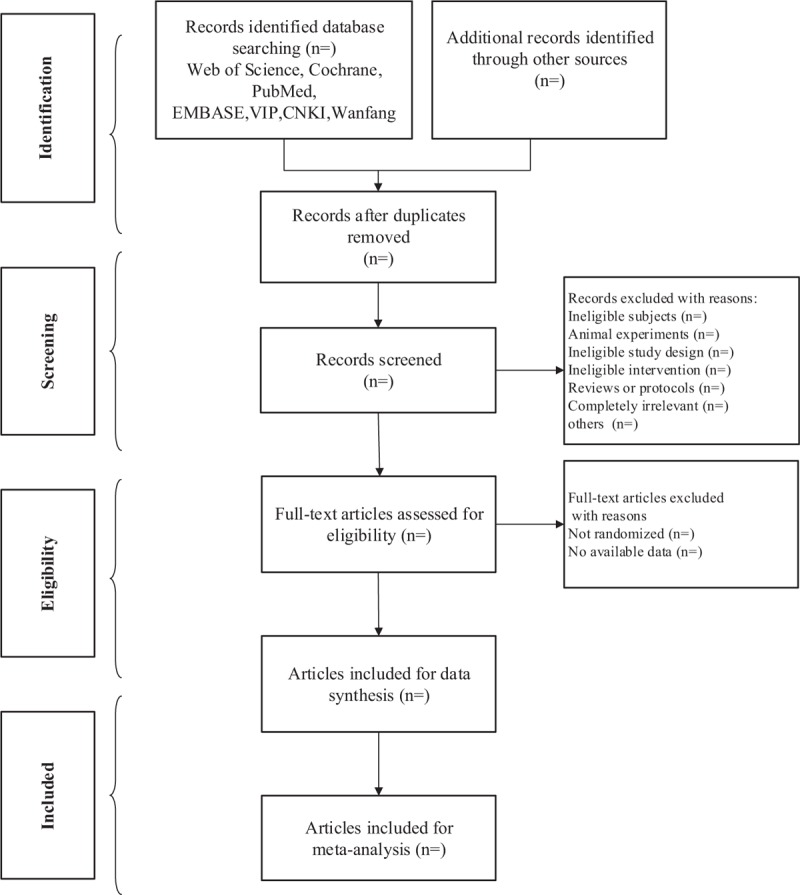
The PRISMA flow chart of selection process.

The researchers will import the retrieved literature into an EndNote library and eliminate duplicate data. Two reviewers (CJ, YYL) will scan the titles and abstracts of all collected articles and will delete articles that are noticeably below-standard. Next, the same 2 reviewers (CJ, YYL) will evaluate the full texts independently, contact the author for research details and discuss the studies to determine the final inclusion of the literature. When the 2 reviewers cannot agree on the selection process through consultations, a third independent reviewer (ZHL) will serve as an arbitrator and ultimately make the decision. The primary selection process is shown in a PRISMA flow chart (Fig. 1).

#### 2.5.2 Data extraction and management

Data from the selected eligible articles will be extracted by 2 independent reviewers and entered into an Excel form. The extracted information includes the reference ID, name of the first author, time of publication, country, participant characteristics, intervention, sample size, blinding, randomization, outcome measures, duration of follow-up, adverse effects, and other detailed information. If necessary, we will contact the corresponding authors of trials as much as possible for further information.

#### 2.5.3 Assessment of risk of bias and reporting of study quality

Two review authors will independently evaluate the risk of bias to evaluate the quality of the studies using the Cochrane Collaboration's risk-of-bias assessment method. The following domains will be accessed for risk of bias: random sequence generation, allocation concealment, blind method for patients, researchers and outcome evaluators, incomplete result data, selective reporting, and other issues. The risk of bias will be assessed and classified according to 3 levels: low risk, unclear risk, and high risk. Any discrepancies will be resolved through discussions and negotiations with the third author. When a consensus on the risk assessment cannot be reached by discussion, the third reviewer will make the decision.

#### 2.5.4 Measures of treatment effect

For continuous data, if the results are measured on the same scale, we will calculate the mean difference (MD) and 95% confidence interval (CI). Standardized MDs with 95% CIs will be used if specific outcome metrics are measured using different outcome measurement scales. The relative risk (RR) will be used to evaluate the enumeration data.

#### 2.5.5 Management of missing data

If there are missing or incomplete data for the primary results, we will attempt to contact the corresponding authors of the referenced articles for the missing data by email. However, if we do not receive a response from the authors for more than a month, then that study will be excluded from the analysis.

#### 2.5.6 Assessment of heterogeneity

Review Manager (RevManV.5.3.5 for Windows; the Nordic Cochrane Centre, Copenhagen, Denmark) will be applied to assess the curative effect and publication bias.

According to the Cochrane Handbook for Systematic Reviews of Interventions,^[26]^ heterogeneity can be evaluated by the *I*^*2*^ statistic and *χ*^*2*^ test.^[27]^ We will use *I*^*2*^ statistics to assess the heterogeneity, and heterogeneity will be considered substantive if *I*^*2*^ > 50%. A forest plot will be used to illustrate the relative strength of the curative effect. Furthermore, the funnel plot will picture the publication bias visually if the number of trials is more than 10. If significant heterogeneity is detected, we will conduct a meta-analysis of these research results using a random-effects model.

#### 2.5.7 Assessment of reporting biases

If more than 10 trials are included in the study, the visual asymmetry on the funnel plot will be used to evaluate the reported bias. If funnel plot asymmetry is detected, the reasons for this outcome will be analyzed.

#### 2.5.8 Data synthesis

RevManV.5.3.5 software will be used for all statistical analyses. We will decide to use either a fixed-effects model or a random-effects model based on the heterogeneity levels of the included studies. If there is no statistical heterogeneity among the results, the fixed effects model will be employed for the meta-analysis. If there is considerable heterogeneity, we will use a random-effects model with 95% CIs to analyse the pooled effect estimates.

#### 2.5.9 Subgroup analysis

If there is significant heterogeneity in the included trials, we will conduct a subgroup analysis based on the control intervention and different outcomes.

#### 2.5.10 Sensitivity analysis

We will conduct a sensitivity analysis to identify whether the conclusions are robust in the review according to the following criteria: sample size, heterogeneity qualities, and statistical model (random-effects or fixed-effects model).

#### 2.5.11 Grading the quality of evidence

We will evaluate the quality of evidence by the Grading of Recommendations

Assessment, Development and Evaluation (GRADE) and will rate the quality by the following levels: very low, low, moderate, or high 4 levels.^[28]^

## 3 Discussion

LDH is a common cause of lumbocrural pain and activity limitations in young and and middle-aged people.^[29]^ Herniated lumbar discs usually cause LBP and/or sciatica (radicular leg pain), which is caused by physical pressure, chemical stimulation by inflammatory reactions, microcirculatory disturbances, or nerve root oedema at the level of the herniated nucleus pulposus.^[30]^ LDH has imposed a heavy burden on individuals, families, and society.^[31]^ Acupotomy treatment for LDH is a miniature surgery with higher acceptability and less pain.^[20]^ Although some trials have shown that acupotomy can effectively reduce the symptoms of LDH,^[22,32,33]^ its efficacy has not been evaluated scientifically or systematically. To the best of our knowledge, there are no systematic reviews or meta-analyses of the effectiveness of acupotomy on LDH that have been published. It is crucial to determine whether acupotomy is a good option in LDH patients and whether it is as effective as other conservative therapies. The aim of this study is to evaluate the efficacy and safety of acupotomy treatment in patients with LDH. Our systematic review and meta-analysis will provide important information to benefit patients, clinicians and health policy-makers with a deeper understanding of the effectiveness of acupotomy therapy.

## Author contributions

ZHL is the guarantor of the article. The article was drafted by CJ and ZHL. CJ, XML, and YYL developed the search strategy. CJ and YYL will independently screen the potential studies and extract the data. CJ and YYL will assess the risk of bias and finish the data synthesis. ZHL will arbitrate any disagreement and ensure that no errors occur during the review. All review authors critically reviewed, revised, and approved the subsequent and final version of the protocol.

**Conceptualization:** Cai Jiang, Yinyan Li.

**Data curation:** Cai Jiang, Yinyan Li.

**Formal analysis:** Cai Jiang, Xiaomei Li.

**Funding acquisition:** Cai Jiang.

**Investigation:** Cai Jiang, Yinyan Li, Xiaomei Li, Shengxian Yu.

**Methodology:** Cai Jiang, Yinyan Li, Zhonghua Lin.

**Project administration:** Cai Jiang, Zhonghua Lin.

**Software:** Miaomiao Guo, Jinhua Guo.

**Supervision:** Miaomiao Guo, Xiaomei Li, Jinhua Guo.

**Validation:** Yinyan Li, Miaomiao Guo.

**Visualization:** Shengxian Yu, Miaomiao Guo.

**Writing – original draft:** Cai Jiang, Yinyan Li, Zhonghua Lin.

**Writing – review & editing:** Cai Jiang, Zhonghua Lin.

## Supplementary Material

Supplemental Digital Content
