# Supplementary material for: Acupotomy therapy for lumbar disc herniation: Protocol for a systematic review and meta-analysis
Source: Medicine (Baltimore). 2018 Oct 5;97(40):e12624. doi: 10.1097/MD.0000000000012624 (PMC6200472; doi:10.1097/MD.0000000000012624)
Supplement: Supplemental Digital Content [file medi-97-e12624-s001.docx]

Acupotomy therapy for lumbar disc herniation: protocol for a systematic review and meta-analysis

**Appendix 1** Search Strategy for Pubmed:

#1 "Single-Blind Method"[Mesh] OR "Double-Blind Method"[Mesh] OR "Randomized Controlled Trials as Topic"[Mesh] OR "Randomized Controlled Trial" [Publication Type] OR "Intention to Treat Analysis"[Mesh] OR "Controlled Clinical Trials as Topic"[Mesh] OR "Clinical Trials as Topic"[Mesh] OR "Clinical Trial" [Publication Type]

#2 "random*"[Text Word] OR allocation[Text Word] OR "random allocation"[Text Word] OR placebo[Text Word] OR single blind[Text Word] OR double blind[Text Word] OR "randomized controlled trial*"[Text Word] OR RCT[Text Word]

#3 randomized controlled trial[Publication Type]

#4 #1 OR #2 OR #3

#5 animals NOT humans

#6 #4 NOT #5

#7 acupotomy [Text Word] OR " small needle-knife "[Text Word] OR needle knife [Text Word]

#8  herniated disc [Title/Abstract] OR " herniated disk *"[Title/Abstract] OR disc prolapse [Title/Abstract] OR disk prolapses [Title/Abstract] OR disc prolapses [Title/Abstract] OR " disk prolapse "[Title/Abstract] OR intervertebral disc displacement* [Title/Abstract] OR " intervertebral disk displacement* " OR " slipped disc " OR " prolapsed disc " OR " prolapsed disk "

#9 #6 AND #7 AND #8
